# COVID-19 FAQs in Pediatric Cardiac Surgery

**DOI:** 10.1177/2150135120924653

**Published:** 2020-04-21

**Authors:** Emily Levy, Jennifer Blumenthal, Kathleen Chiotos, Joseph Dearani

**Affiliations:** 1 Divisions of Pediatric Infectious Diseases and Pediatric Critical Care Medicine, Department of Pediatric and Adolescent Medicine, Mayo Clinic, Rochester, MN, USA; 2 Divisions of Critical Care Medicine, Department of Anesthesiology, Critical Care and Pain Medicine, Division of Infectious Diseases, Department of Medicine, Boston Children’s Hospital, Boston, MA, USA; 3 Divisions of Infectious Diseases and Critical Care Medicine, Children’s Hospital of Philadelphia, Philadelphia, PA, USA; 4 Department of Cardiovascular Surgery, Mayo Clinic, Rochester, MN, USA

**Keywords:** COVID-19, congenital heart disease, frequently asked questions, education

## Introduction

The world at large and the United States’ health care infrastructure face unprecedented challenges in the COVID-19 pandemic, and the congenital heart disease (CHD) community is no exception. These challenges include potential resource scarcities of equipment, personnel, and blood products. There is also a potential risk of infection to healthcare providers and family members. The relatively small size of the CHD workforce adds another dimension to the challenge, since the rapid spread of COVID-19 could result in programmatic collapse at a moment’s notice secondary to insufficient personnel from infection or quarantine. While many segments of our culture can pause during this period of crisis, pediatric patients’ diseases require continuing care, particularly amongst newborns and infants who often require surgery during a narrow window of time to avoid death and provide for optimal outcomes. The medical community has been overwhelmed with video conferences, webinars and newsletter updates that have covered a broad range of topics. Many of the questions now center on critical care and infectious disease (ID) related issues – screening techniques, preventative measures, treatment options, etc. Crisis management strategies for congenital heart disease have recently been published^1^. The purpose of this review is to succinctly summarize frequently asked questions related to COVID-19 as it relates to children with congenital heart disease.

1. What is the role of nasal swab versus serum testing in a child needing heart surgery?

The best test for SARS-CoV2 in the peri-operative setting is a PCR of respiratory secretions. These samples may be from a nasopharyngeal swab, oropharyngeal swab, or sample from the lower respiratory tract (e.g., tracheal aspirate or BAL), if available at your center. PCR testing sensitivity is dependent on viral SARS-CoV2 concentrations at the site of the sample, thus it may be affected by sampling technique, by progression of the disease, and by the test itself. As the disease progresses, viral load tends to decrease in the upper respiratory tract. Serology serum testing for antibodies (IgG) may be available in some centers. However, positive serology will demonstrate prior exposure (or maternal status for neonates) rather than active illness, so is less useful in a peri-operative setting.

https://www.cdc.gov/coronavirus/2019-nCoV/index.html ^2^

2. What is the optimal pre-operative testing? Is there a role for CT chest scan to look at lungs the day before surgery?

If pre-operative testing is used, PCR-based testing of respiratory secretions is the most widely accepted approach. All patients should additionally be screened for symptoms. There are reports demonstrating chest CT abnormalities in adults during asymptomatic/pre-symptomatic disease; however, the role of chest CT in relation to COVID-19 in children remains undifferentiated at this time. Given lack of evidence, radiation exposure, and potential sedation-requirement in younger children, CT scans should not be used to screen for or diagnose pediatric COVID-19. CT scans should be reserved for other clinical indications based on symptoms.

Fu L, Wang B, Yuan T, Chen X, et al. Clinical characteristics of coronavirus disease 2019 (COVID-19) in China: a systematic review and meta-analysis [published online ahead of print April 15, 2020]. *J Infect.* Doi: 10.1016/j.jinf.2020.03.041. Accessed April 17, 2020.^3^

3. Should parents of pediatric cardiac patients be screened?

All parents entering the hospital or clinic should be screened for symptoms suggesting COVID-19 (including cough or fever) as well as for contact with known positive cases. Whether microbiologic test screening is performed routinely depends on a multitude of factors, including local prevalence, whether parents are required to mask in the hospital, and testing availability at that center. Parents of cardiac patients should follow local guidance in accordance with the CDC guidance.

https://www.cdc.gov/coronavirus/2019-nCoV/index.html ^2^

4. What is the best approach for a newborn of COVID positive mother who will need heart surgery in the first 1-2 weeks of life (e.g., arterial switch, Norwood, etc.)?

Consensus guidelines for infants born to mothers with COVID-19 are not yet finalized, though are in process. To date, there is minimal evidence of placental vertical transmission, so we suspect most maternal to neonatal transmission occurs at birth or shortly after via droplet contamination. All infants born to COVID positive mothers should be considered persons under investigation (PUI). It may be reasonable to separate the infant from the mother if the infant will need cardiac surgery in order to try and avoid post-natal infection. It may also be reasonable to do serial testing on the infant; some experts recommend at 2, 4, and 6 days of life while others recommend at 24-48 hours of life and then again at 14 days of life. This should be done in conjunction with consultation from local experts.

https://downloads.aap.org/AAP/PDF/COVID%2019%20Initial%20Newborn%20Guidance.pdf ^4^

Schwartz DA. An Analysis of 38 Pregnant Women with COVID-19, Their Newborn Infants, and Maternal-Fetal Transmission of SARS-CoV-2: Maternal Coronavirus Infections and Pregnancy Outcomes [published online ahead of print March 17, 2020]. *Arch Pathol Lab Med.* doi: 10.5858/arpa.2020-0901-SA. Accessed April 17, 2020.^5^

5. What factors determine optimal timing of surgery in patients who have been COVID-19 positive?

There is no evidence to suggest optimal timing of surgery in COVID-19 positive patients. Surgery should be scheduled with advice from a multidisciplinary team of experts including cardiac medical, cardiac surgical, and infectious diseases as indicated. If prudent, surgery should be delayed until the patient’s symptoms have improved and/or testing has been repeated (often after 14 days) and is negative.

https://www.cdc.gov/coronavirus/2019-ncov/hcp/disposition-hospitalized-patients.html ^6^

6. Can an asymptomatic COVID-19 positive child transmit the virus?

Yes, asymptomatic patients may shed virus and may transmit virus.

https://www.cdc.gov/coronavirus/2019-nCoV/index.html ^2^

7. Which CHD population should we worry about most? Which pediatric patients are at highest risk?

In comparison to younger children, teenagers and adults are at higher risk for morbidity and mortality, with particular risk factors including pre-existing conditions (most notably diabetes, obesity, and hypertension), immunocompromised, and advanced age. Thus, adult congenital heart patients and the post-repair cardiac population may be at highest risk of severe disease.

Children have had mild disease in general, though we do not yet have data specifically about infants with congenital heart disease. There are some case series to suggest higher frequency of pediatric COVID-19 cases in infants <1 year of life compared with older children, but data are extremely limited.

Cruz AT, Zeichner SL. COVID-19 in Children: Initial Characterization of the Pediatric Disease. *Pediatrics*. 2020;145(6): e20200834.^7^

8. Is there a role for antiviral therapy?

The recommended treatment for COVID-19 is supportive care. In some critically ill children, there may be a role for specific antiviral therapy, though there are no compelling data supporting efficacy of any available antiviral agent at the time of publication.

Remdesivir is an antiviral being studied in adults in several RCTs and may be used in children through single patient expanded access requests. Hydroxychloroquine has been used, but there are increasing safety concerns related to risk of QTc prolongation and still extremely limited efficacy data. Extreme care should be taken in patients at increased risk of prolonged QTc. There may also be a role for convalescent plasma (plasma from patients who have had COVID-19 and now have antibodies) during pediatric critical illness; this therapy is still being studied. The use of corticosteroids is not recommended as it may prolong viral replication.

https://www.nature.com/articles/d41573-020-00016-0 ^8^

Wang M., Cao R., Zhang L., Yang Z., et al. Remdesivir and chloroquine effectively inhibit the recently emerged novel coronavirus (2019-nCoV) in vitro. *Cell Research*. 2020;30: 269-271.^9^

https://www.medrxiv.org/content/10.1101/2020.04.10.20060699v1 ^10^

https://www.medrxiv.org/content/10.1101/2020.04.02.20047050v1.article-info ^11^

https://www.fda.gov/vaccines-blood-biologics/investigational-new-drug-ind-or-device-exemption-ide-process-cber/recommendations-investigational-covid-19-convalescent-plasma ^12^

9. Is there a role for therapeutic plasma exchange?

Plasma exchange is unlikely to be useful as the major reservoirs for this virus are respiratory tract and GI tract.

10. What are potential strategies to mitigate the reported IL-6 cytokine storm?

Tocilizumab is an anti-IL6 therapy which is used in cytokine storms associated with certain oncologic therapies (for instance, CAR-T therapy). It is hypothesized that it may be of benefit for patients with COVID-19 induced cytokine storm. If used, its use should be reserved for those with high levels of IL-6 and use should be in consultation with a multidisciplinary team including pediatric infectious diseases, pediatric critical care, and pharmacy representatives. Use of tocilizumab may place patients at risk for subsequent opportunistic infections and potentiate a sepsis-like syndrome.

Luo P, Liu Y, Qiu L, Liu X, Liu D, Li J. Tocilizumab treatment in COVID-19: a single center experience [published online ahead of print April 8, 2020]. *J Med Virol.* doi: 10.1002/rmv.2107. Accessed April 17, 2020.^13^

## Summary

Unprecedented times call for unprecedented measures and elite teamwork. Prioritization and appropriate timing of surgery are essential. Continuous collection of evidence during the COVID-19 crisis will help guide decision-making, particularly with regard to critical care and ID-related issues. Practical guidance strategies include ensuring safety and tactics for children with CHD, their families, and all of the healthcare providers involved in their care. The pediatric cardiac surgery team has been marked by unanimity and camaraderie. This cohesive team carries a history notable for collaboration, flexibility, adaptation, and immediate readiness. This assortment of FAQ’s and expert answers is yet another constructive strategy to improve pediatric patient care in this time of crisis.
